# Correlation between helicobacter pylori infection and iron deficiency in children

**DOI:** 10.12669/pjms.38.5.5175

**Published:** 2022

**Authors:** Yuanda Zhang, Jing Bi, Meiying Wang, Hongyu Deng, Wenli Yang

**Affiliations:** 1 Yuanda Zhang, Department of Nutrition, Baoding Key Laboratory of Clinical Research on Children’s Respiratory and Digestive Diseases, Baoding, Hebei, 071000, P.R. China; 2 Jing Bi, Department of Infectious Diseases, Baoding Laboratory for Accurate Diagnosis and Treatment of Pediatric Infectious Diseases, Baoding, Hebei, 071000, P.R. China; 3 Meiying Wang, Department of Clinical Laboratory, Baoding Children’s Hospital, Baoding, Hebei, 071000, P.R. China; 4 Hongyu Deng, Department of Clinical Laboratory, Affiliated Hospital of Hebei University 071000, China; 5 Wenli Yang, Department of Clinical Nutrition, Beijing Children’s Hospital, Capital Medical University, National Children’s Medical Center, Beijing 100045, China

**Keywords:** Children, Helicobacter pylori, Iron deficiency, Questionnaire

## Abstract

**Objectives:**

To investigate the correlation between Helicobacter pylori (Hp) infection and Iron deficiency (ID) in children.

**Methods:**

This cross-sectional study was conducted at Baoding Children’s Hospital from January 2018 to December 2019. A total of one thousand children who came to our hospital for physical examination and met the inclusion criteria were continuously included in this study. All the children were given questionnaires (personal and family social and economic status), a stool Hp antigen test and/or a 13C-urea breath test, as well as measurements of hemoglobin (Hb), mean red blood cell volume (MCV), red blood cell distribution width (RDW), mean corpuscular hemoglobin concentration (MCHC), serum ferritin (SF), serum iron (SI), and total iron binding capacity (TIBC). Children who tested positive for Hp were divided into the Hp group and children who tested negative for Hp were divided into the control group. ID or IDA was diagnosed based on the child’s blood test results.

**Results:**

A total of 902 children met the inclusion criteria, including 194 (21.5%) in the Hp group and 708 (78.5%) in the control group. The incidence of ID and IDA in Hp group was higher than that in control group (2=9.112, 2=4.478; All P < 0.05); The levels of MCV, SI, SF and Hb in Hp group were lower than those in control group (t=5.288; T = 3.864; T = 6.751; T =11.841, all P < 0.05), TIBC level was higher than that of control group (T =7.630, P < 0.05); The levels of MCHC and RDW in THE Hp group were not statistically significant compared with the control group. Logistic regression showed that Hp infection was not a combined risk factor for ID. Older age, higher educational background of the mother, living in the city, and higher family income were the combined protective factors to prevent the occurrence of ID in children.

**Conclusion:**

Hp infection is not a combined risk factor for the development of ID in children. The influence of family social and economic factors should be taken into consideration when analyzing the correlation between Hp infection and ID.

## INTRODUCTION

Many children in China are infected with Helicobacter pylori (Hp). The cause of Hp infection in children may be related to close contact with parents, which means they can get it from an infected adult.^1^ It has been shown in some studies that Hp infection can not only cause digestive system diseases, but also has a close bearing on iron-deficiency anemia.^2^ Iron deficiency (ID) has a high incidence in children in China,^3^ and may develop into iron deficiency anemia (IDA) if there is no timely intervention. It is considered in most studies that Hp infection is associated with ID,^4^ and patients with Hp infection may suffer from iron deficiency due to occult blood loss or reduced iron absorption.^5^

Nevertheless, different research groups have different conclusions, and some studies believe that Hp infection has nothing to do with ID.^6^,^7^ When discussing the correlation between Hp infection and ID, some factors other than Hp should be considered. It is currently believed that the Hp infection rate increases with age and is affected by social and economic factors.^8^ The incidence of ID is negatively correlated with age and positively correlated with socioeconomic factors.^9^,^10^ Therefore, age, family, social and economic factors should be included in the discussion of the correlation between Hp infection and ID. In order to clarify whether there is a correlation between Hp infection and ID in children in this area, in this study, Hp infection, ID and IDA indicators were examined in the enrolled children, and information on social and economic situation of individuals and their families was collected through a questionnaires to further explore whether Hp infection in children is a risk factor for ID.

## METHODS

This is a cross-sectional study. A total of one thousand children who underwent physical examination in Baoding Children’s Hospital from January 2018 to December 2019 were consecutively included, of whom nine hundred two met the inclusion criteria and were enrolled as subjects. In this study, children with confirmed Hp infection were first selected as the Hp group (case group) and those without Hp infection as the control group, and their data were analyzed. Subsequently, univariate analysis was performed on the questionnaire results of 902 enrolled children based on the occurrence of ID, and the factors P<0.05 in chi-square analysis were included in Logistic regression model for multivariate analysis. All enrolled children had obtained the consent of their legal guardians and signed informed consent Forms.

### Ethical Approval:

The study was approved by the Institutional Ethics Committee of Baoding Children’s Hospital on September 15, 2019 (No. [2019]098), and written informed consent was obtained from all guardians of participants.

### Inclusion criteria:

Children aged 4-16 years; Children who were able to undergo a stool Hp antigen test and/or a 13C-urea breath test and have obtained the consent of their guardians. Exclusion criteria: Children who have been taking iron supplements or drugs that affect iron metabolism within six months; Children with severe heart, liver, kidney, lung, nerve, mental, tumor, allergic disease, genetic metabolic disease and other diseases; Children with chronic inflammatory disease or a history of acute infectious disease within the last one month; Children with anaemia from causes other than iron deficiency anaemia; Children with chronic intestinal disease.

### Diagnostic criteria:

***(1) Diagnostic criteria for Hp:*** Children with positive endoscopic pathological staining, rapid urease test, and/or 13C-urea breath test were diagnosed with Hp infection.^11^

***(2) Diagnostic criteria for iron deficiency (ID):*** According to the WHO definition^12^, SF<12μg/L for children younger than 5 years old, and SF<15μg/L for older than five years old.

***(3) Diagnostic criteria for IDA:*** Under the diagnostic criteria of iron deficiency, hemoglobin <110 g/L for children younger than five years old, hemoglobin <115 g/L for 5-11 years old, and hemoglobin <120 g/L for 12-16 years old.

All the 902 enrolled children were subjected to serum inspection of indexes such as hemoglobin (Hb), mean corpuscular volume (MCV), red cell distribution width (RDW), mean corpuscular hemoglobin concentration (MCHC), serum ferritin (SF), serum iron (SI), total iron binding capacity (TIBC), stool Hp antigen detection and/or 13C-urea breath test. For children whose guardians have agreed to have them undergo electronic gastroscopy, we have arranged for a qualified professional to complete the endoscopy. All the guardians of the included children were surveyed by trained professionals, which included personal information of the children and socioeconomic status of the family (gender, age, education level of the mother, place of residence, economic income level, etc.). All specimen collection and test operations were carried out by dedicated personnel with relevant qualifications.

### Specimen Collection:

2 ml venous blood was extracted from one purple cap tube and one red cap tube on an empty stomach. Red cap tube blood was kept at 4°C-20°C for 30min at 3000r/min, centrifuged for 10min, and serum was collected for detection.

### Routine Blood Test:

Mindray BC-5300 automatic blood analyzer was used for the determination. SF, SI and TIBC were determined by chemiluminescence method and were detected by BECKMAN Coulter-AU5800 biochemical analyzer in Baoding Key Laboratory of Clinical Research on Respiratory and Digestive Diseases in Children. SI and TIBC reagents were purchased from BECKMANCOULTER, while SF reagents were sourced from Abbott Laboratories. Gastroscopy was performed by Olympus Q290 electronic gastroenteroscopy, and the examination was performed by a qualified person.

### Statistical Analysis:

All data were analyzed by SPSS25.0 statistical software. Chi-square test was used to compare the rate of classified variables between groups. Continuous variables were represented as “mean ± standard deviation”, and mean comparison between groups was performed using the T test. Multivariate analysis of risk factors for children with ID was performed using logistic regression model. P <0.05 indicates a statistically significant difference.

## RESULTS

One hunded ninety four patients (21.5%) were divided into the Hp group, including 119 males (61.3%) and 75 females (38.7%), aged 8.4±3.2 years. Seven hundred eight cases (78.5%) were divided into the control group, including 428 males (60.5%) and 280 females (39.5%) with an age of 8.7±3.3 years. No statistically significant difference can be seen in the comparison of gender and age between the two groups (P>0.05).Among the 194 patients in the HP group, 34 cases (17.5%) were associated with ID and 12 cases (6.2%) with IDA. Among 708 cases in the control group, 69 cases (9.7%) were combined with ID and 21 cases (3.0%) with IDA. There were 103 cases of ID (11.4%) and 33 cases of IDA (3.7%) in the two groups. The incidence of ID and IDA in the Hp group were higher than those in the control group, with a statistically significant difference (P<0.05). See Table-I for the results.

**Table I:** Prevalence of ID and IDA in the two groups.

Group	Number of cases	IDA	ID
Hp group	194	12(6.2)	34(17.5)
Control group	708	21(3.0)	69(9.7)
χ^2^		4.478	9.112
P value		0.034	0.003

***Note:*** The data in () are expressed as a percentage.

The levels of MCV, SI, SF, and Hb in the Hp group were lower than those in the control group (P<0.05), and the TIBC level was higher than that in the control group (P<0.05); No statistically significant difference can be seen in the comparison of MCHC and RDW levels in the Hp group with the control group (P>0.05), Table-II.

**Table II:** Comparison of blood routine indexes, SI, TIBC and SF between the two groups.

Group	Number of cases	MCV (fl)	MCHC (g/L)	RDW (%)	SI (μmoL/ L)	TIBC (μmoL/ L)	SF (μg/ L)	Hb (g/L)
Hp Group	194	77.6±7.2	323.4±11.9	10.3±1.3	12.3±2.3	71.6±12.7	24.6±10.3	122.4±8.1
Control group	708	80.5±6.9	324.4±10.3	10.5±1.3	13.1±2.6	64.2±13.1	29.9±9.4	130.5±9.5
t value		5.138	1.073	1.155	3.889	7.630	6.751	11.841
P value		0.00	0.284	0.249	0.000	0.00	0.00	0.00

Single factor Chi-square analysis was conducted on Hp infection, age, mother’s education level, residence, family economic income and other factors, respectively. The results showed that Hp infection, mother’s education level, residence, family economic income (P<0.05) were the influential factors for the occurrence of ID in children. The factors of P<0.05 in the chi-square analysis were incorporated into the logistic regression model for multivariate analysis. The results showed that Hp infection was not a combined risk factor for child ID (P>0.05); Older age, higher maternal education and living in urban areas, and higher family income were the combined protective factors for the prevention of ID in children. Table-III-IV for details.

**Table III:** Univariate analysis of individual and family social and economic situation of children with ID.

Related factors	Regression coefficients	Standard error	χ2	P value	OR value	95%CI
HP infection	0.677	0.227	8.861	0.003	1.968	1.260-3.073
Age	-0.513	0.062	67.983	0.000	0.599	0.530-0.677
Higher maternal education	-0.930	0.282	10.840	0.001	0.395	0.227-0.686
Living in urban areas	-1.613	0.400	16.292	0.000	0.199	0.091-0.436
Better economic conditions	-2.217	0.296	55.934	0.000	0.109	0.061-0.195

**Table IV:** Multi-factor analysis of individual and family social and economic situation of children with ID.

Related factors	Regression coefficients	Standard error	χ2	P value	OR value	95% CI
HP infection	0.132	0.266	0.246	0.620	1.141	0.678-1.920
Age	-0.499	0.066	57.613	0.000	0.607	0.533-0.690
Higher maternal education	-0.698	0.312	5.009	0.025	0.498	0.270-0.917
Living in urban areas	-1.030	0.439	5.507	0.019	0.357	0.151-0.844
Better economic conditions	-1.839	0.322	32.631	0.00	0.159	0.085-0.299

## DISCUSSION

Iron deficiency (ID) and iron deficiency anemia (IDA) may affect children’s neurocognitive development and immune function, and have a significant impact on children’s health.^10^,^12^,^13^ It is negatively correlated with age in terms of morbidity.^10^,^14^ It has been reported that the incidence of IDA in Chinese children aged 3-5 and 6-18 was 10.4% and 8.0%, while the incidence of IDA was 2.9% and 2.8%,^15^ which was consistent with the incidence of ID (11.4%) and IDA (3.7%) in children aged 4-16 years in this study.

Different research groups have different conclusions about the correlation between Helicobacter pylori (Hp) infection and ID and IDA in children. In this study, a Logistic regression model was carried out to analyze the correlation between Hp infection and ID and IDA in children.

Hp infection has been associated with ID and IDA in children in some studies.^10^,^16^ Hp infection may cause iron deficiency by virtue of gastrointestinal blood loss, reducing the absorption of dietary iron, and enhancing the absorption of iron by bacteria.^17^ It has also been reported that Hp infection has no correlation with the incidence of ID and IDA in children,^6^,^18^ that Hp infection alone might not be sufficient to cause IDA,^19^ and socioeconomic factors should be considered when analyzing the impact of Hp infection on the prevalence of ID in children.^20^ In this study, the prevalence of ID and IDA in Hp-infected and non-HP-infected children was compared in univariate analysis using the Chi-square test. The results showed that the prevalence of ID and IDA in Hp-infected children aged 4-16 was higher than that in the control group. Subsequently, it was found that Hp infection was not a combined risk factor of ID when the significant chi-square test factors were selected to further analyze the risk factors of ID in children via Logistic regression model, suggesting that Hp infection may not be directly correlated with ID.

A prospective, cross-sectional and population study conducted in three provinces and cities in China suggested that the Hp infection rate of children in China was related to the economy, and that high maternal education, large family living area and living in urban areas had a protective effect on preventing Hp infection in children.^8^ High maternal education, living in urban areas and high family income were important protective factors to prevent ID.^21^ As shown in the results of this study, higher maternal education, living in urban areas, and higher family income are the combined protective factors to prevent the occurrence of ID in children. Better family social and economic conditions are considered to be protective factors for Hp infection, ID and IDA.

### Limitations:

Nevertheless, some limitations are still visible in this study like lengthy enrollment time, single-center study, and no further analysis of children with the same family social and economic factors.

## CONCLUSION

The family social and economic conditions of children in the Hp group in study lag behind those of children without Hp infection, that is, the control group. Therefore, the chi-square test of the prevalence of ID and IDA in children in the two groups will provide meaningful results. In view of this, care should be taken to exclude the influence of family, social, economic and other factors when discussing whether the prevalence of ID in children with Hp infection is higher than that of children without Hp infection.

### Authors’ Contributions:

**YZ &** **JB:** Designed this study and prepared this manuscript, are responsible and accountable for the accuracy or integrity of the work.

**WY:** Collected and analyzed clinical data.

**MW &** **HD:** Significantly revised this manuscript.
